# Food protein induced enterocolitis syndrome to fish: a report of 20 cases

**DOI:** 10.1186/2045-7022-5-S3-P55

**Published:** 2015-03-30

**Authors:** Ioanna Vasilopoulou, Efstratia Charitidou, Maria Trigka

**Affiliations:** 1 Department of Paediatrics, Paediatric Allergy Unit, University General Hospital of Patras, Patras, Greece; 2 Department of Mathematics, School of Applied Mathemathical and Physical Sciences, National Technical University of Athens, Athens, Greece

## Introduction

Food protein induced enterocolitis syndrome (FPIES) is non-IgE mediated disorder triggered by the ingestion of certain food proteins. The diagnosis of FPIES is frequently delayed because of non-specific symptoms and lack of biomarkers.

## Objective

To describe the clinical characteristics and the natural evolution of FPIES provoked by fish in children.

## Methods

We retrospectively studied the medical records of patients evaluated in our practice over a 9-year period.

## Results

Sixteen children (11 male) experienced 47 episodes of FPIES caused by fish. Mean age of onset was 12.3 months (SD 2.2). Among symptoms, vomiting was dominant (100%) while diarrhea was less frequently observed (9%). Only 5/47 cases (11%) were admitted to the emergency unit. None of these children presented with symptoms of hypovolemic shock. Mean time of symptom onset after ingestion was 2.4 h (SD 0.7). The median number of prediagnosis reactions was 3 episodes (range 2-5). Skin prick tests to fish allergen were negative in all cases. Among children, 5 had atopic dermatitis and/or IgE sensitization to egg and 1 had allergic proctocolitis. 7/16 children (44%) had positive atopic family history. Concerning birth data 9/16 (56%) were born after cesarean section and the majority (94%) was full term. 7/16 children (44%) were exclusively breastfeeding for at least 4 months. We also report another 4 children (2 male) who presented after one typical episode of FPIES after fish ingestion but parents denied the oral food challenge in order to confirm the diagnosis. Data on the evolution of the syndrome are available at the moment only on 7 children. At a median age of 4 years (range 2.5 - 5.5 y) resolution was observed in none of the cases. At the age of 9, data exist for 2 children; one became tolerant while the other failed to outgrow the symptoms.

## Conclusions

Our data suggest that the clinical presentation of FPIES due to fish is less severe compared to other foods, especially milk. Moreover it appears that the clinical course of the syndrome is more protracted.

